# Spontaneous tension haemopneumothorax

**DOI:** 10.1186/1757-7241-16-12

**Published:** 2008-10-31

**Authors:** Benjamin Oliver Patterson, Sarah Itam, Fey Probst

**Affiliations:** 1 Department of Emergency Medicine, Charing Cross Hospital, Fulham Palace Road, W6 8RF, London

## Abstract

We present a patient with sudden onset progressive shortness of breath and no history of trauma, who rapidly became haemodynamically compromised with a pneumothorax and pleural effusion seen on chest radiograph. He was treated for spontaneous tension pneumothorax but this was soon revealed to be a tension haemopneumothorax. He underwent urgent thoracotomy after persistent bleeding to explore an apical vascular abnormality seen on CT scanning. To our knowledge this is the first such case reported.

Aetiology and current approach to spontaneous haemothorax are discussed briefly.

## Introduction

Spontaneous haemopneumothorax (SHP) occurs in 1–12% cases of spontaneous pneumothorax and is characterised by spontaneous pneumothorax with over 400 mls of blood in the pleural cavity [1]. This can go unnoticed or can cause life threatening hypovolaemia.

## Case report

**Figure 1:**
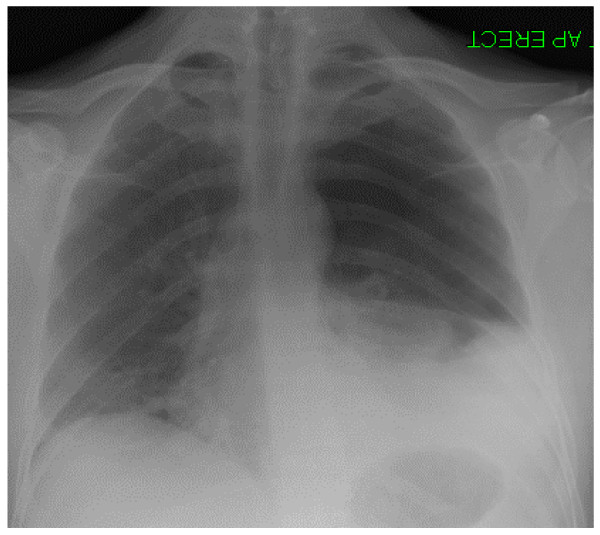
Erect chest radiograph showing pneumothorax occupying upper third of left lung field with some mediastinal shift and basal opacification.

**Figure 2:**
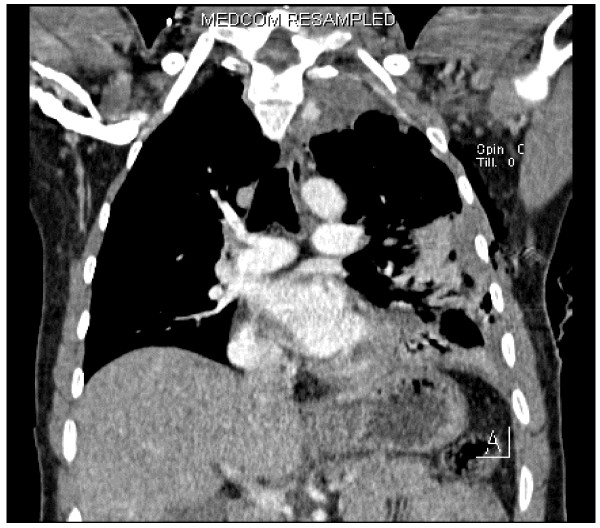
Computerised tomography of the thorax with contrast showing vascular abnormality in the left apical area adjacent to second and third thoracic vertebrae.

Whilst pivoting in his chair at work, a 35 year old man suddenly developed chest pain and difficulty in breathing associated with a "gurgling in the chest". The pain radiated to his left shoulder tip and was worse on deep inspiration. He attended the Emergency Department, and while he was being triaged it was noticed that he was tachycardic with decreased air entry on the right. He was slightly tachypnoeic but oxygen saturations were acceptable on high flow oxygen via a reservoir bag. The working diagnosis was spontaneous pneumothorax, and as he was stable no immediate action was taken. A chest radiograph was requested whilst he awaited formal medical assessment and treatment (fig. 1). This was initially interpreted as an apical pneumothorax with a possible effusion. While this was being reviewed and the significance of the effusion discussed he become very anxious and his blood pressure suddenly dropped from normal to unrecordable. He was tachycardic with a faint, thready pulse and oxygen saturations of 93% despite receiving 15L of oxygen a reservoir bag. His trachea was pushed anteriorly with visible hyperexpansion of the left hemithorax, and he was clammy with rivulets of sweat on his chest. A new diagnosis of tension pneumothorax was made, and the pleural cavity was immediately decompressed with needle thoracostomy followed by prompt insertion of chest drain using the Seldinger technique. This was seen to be functioning well, and within minutes there was an improvement of symptoms, with blood pressure and oxygen saturations restored to normal. A few minutes later he again became anxious with difficulty breathing and complained of feeling dizzy. His blood pressure fell again and he became cold and clammy. A wide bore chest drain was inserted, as it was feared that the original drain was not functioning and the tension pneumothorax was re-accumulating. Immediately 300 mls of bright red blood was measured to drain with 400 mls following over the next ten minutes. Again there was immediate symptomatic improvement and vital signs normalised. A new chest radiograph was obtained and in comparison with the previous film the pleural effusion had worsened. An urgent CT scan revealed a large haemothorax in spite of the drains and a "small focal well circumscribed area of high density in the superior mediastinum on the left side that does not show continuity with any vessel that has an unusual appearance and may represent a small pseudo-aneurysm or sentinel clot" (fig. 2). It was thought unlikely at this point that the haemothorax was iatrogenic due to the presence of effusion before the initial decompression was undertaken. By now 1300 mls of blood had been drained in total from both drains and two units of blood had been transfused, and the patient was transferred to a cardiothoracic centre where he underwent urgent exploratory thoracotomy after video assisted thoracoscopic examination failed to identify the cause. Continued bleeding was discovered in a ruptured apical bulla, and haemostasis was achieved with a combination of diathermy and under-running. Approximately 1000 mls of blood and clot was removed from the left hemithorax with placement of both apical and basal intercostal drain. He was extubated immediately and made a good recovery to be discharged on post-operative day nine with no complications to date.

## Discussion

We could find no reports of spontaneous tension haemopneumothorax in the literature, as defined by a pubmed search using the keywords and MeSH terms "spontaneous haemothorax" and/or "tension pneumothorax". Here SHP is discussed as the underlying pathological process is the same. In a recent review of the management of 211 cases of SHP, it is a described as a condition affecting mostly young men with three main causes. These are bleeding from a torn adhesion between the pleura, rupture of a vascularised bulla or an aberrant apical vessel associated with a bulla that ruptures [2]. Treatment depends on clinical condition and facilities available. A third of patients in the series were shocked and needed urgent surgery, and in a further third it was clear that immediate operation for haemostasis would be required. However nine out of ten patients would eventually need surgery, and indications for delayed surgery were persistent air leak and clot evacuation. Of those having surgery 43% had a video assisted thoracoscopic surgical (VATS) procedure which was definitive, and 45% required a thoracotomy. Post operative results are good with no major complications and no recurrence. The remaining patients were treated conservatively having none of the above indications for surgery. In a small series of nine such cases with mean blood loss of 1533 mls, it was successful in seven but with two needed a thoracotomy due to worsening clinical condition [3]. This author suggests that if bleeding persists for less than 24 hours then conservative treatment is adequate. In a report of four cases of spontaneous tension pneumothorax the diagnosis was made radiologically rather than clinically on every occasion, and in each case the clinicians were not aware that it this could happen [4]. No reports exist of spontaneous pneumothorax presenting under tension.

## Conclusion

Spontaneous tension pneumothorax is very rare, but requires treatment in the same manner as one of any other aetiology. This may also be associated with haemothorax which could be apparent radiologically, or during the course of treatment with an intercostal drain. These patients may develop hypovolaemic shock and most with clinically obvious bleeding require operative intervention. If the patients vital signs are stable enough they should have a CT scan to look for a bleeding point and be observed in an environment where thoracic surgeons can be called upon for assistance. If operation is not required in the first 24 hours then the chances of needing surgery are reduced.

## Competing interests

The authors declare that they have no competing interests.

## Authors' contributions

BP conducted the literature search and partly wrote the case report. SI assisted with the literature review. FP partly wrote the case report and critically appraised the final draft. All authors read and approved the final manuscript.

## Consent

Written informed consent was obtained from the patient for the publication of this case report and accompanying images. A copy of the written consent is available for review by the Editor-in-Chief of this journal
